# Lotilaner - a novel systemic tick and flea control product for dogs

**DOI:** 10.1186/s13071-017-2471-3

**Published:** 2017-11-01

**Authors:** Susan E. Little

**Affiliations:** Center for Veterinary Health Sciences, Oklahoma State University, Stillwater, OK 74074 USA

Safe, effective control of ticks and fleas is critically important for the health and well-being of companion animals and the people with whom they share their lives. Mitigating the risk created by tick and flea infestations protects dogs and humans not only from the arthropods but also, in many cases, from the serious infections they transmit. An added and perhaps equally important benefit of successfully controlling ticks and fleas is protecting the human animal bond. Modern tick and flea control products such as lotilaner, the novel isoxazline described in this special issue, provide veterinarians and pet owners with a simple, reliable strategy to help eliminate these pests from pets and make the distress of home infestations a distant memory. Removing ticks and fleas from the dog-human equation fosters a closer relationship between people and their pets which in turn benefits many aspects of public health, both physical and mental [1]. Supporting the human-animal bond and protecting both canine and human health are some of the primary reasons the Companion Animal Parasite Council (capcvet.org) and the European Scientific Counsel on Companion Animal Parasites (esccap.org) recommend routine tick and flea control for dogs.

This special issue debuts a collection of detailed studies performed to evaluate the safety, efficacy, and performance characteristics of a novel systemic isoxazoline insecticidal and acaricidal compound - lotilaner - developed by Elanco specifically for use in companion animals to quickly address canine tick and flea infestations. The pharmacokinetic and safety studies described document that lotilaner is rapidly absorbed and was not associated with any treatment-related or pathological effects even when elevated doses were administered for several months [2, 3]. The excellent safety profile of lotilaner and other isoxazolines highlights another advantage of using more recently developed insecticides and acaricides in pets. Indeed, products like lotilaner have largely supplanted use of the more toxic, often now-banned, historic compounds such as organochlorines, organophosphates and carbamates [4, 5].

*Ctenocephalides felis* continues to reign as the most common flea associated with pets worldwide. Despite several decades of widely available, sound flea control products, infestations remain a substantial canine health concern, an issue compounded by the presence of insecticide-resistant populations of *C. felis* [6]. The experimental flea infestation studies in this special issue demonstrate that the novel systemic insecticide lotilaner begins killing fleas as soon as 2 h after administration and maintains a rapid (within 4 h) high efficacy against subsequent re-infestations for at least 35 days after initial administration [7, 8]. Field trials confirmed that owners using lotilaner can expect to see elimination of flea infestations and significant reduction of flea allergy dermatitis [9, 10]. Rapid kill, complete elimination of infestations, and prevention of re-infestation are critically important for minimizing the dermatitis associated with allergy to fleas because even a small number of bites can result in recrudescence of clinical signs [11]. Moreover, lotilaner tablets were readily accepted by dogs and, in a comparison field trial, achieved better efficacy against fleas than fipronil [10]. This high acceptability is important - despite the many advances in safety and efficacy of parasite control products, lack of compliance remains a significant barrier to achieving flea control in dogs [12–14].

A diverse array of ticks infest dogs around the world, including *Rhipicephalus sanguineus* (*sensu lato*) wherever dogs are found; *Ixodes ricinus* and *Dermacentor reticulatus* in Europe; and *Amblyomma americanum*, *Dermacentor variabilis* and *Ixodes scapularis* in North America. Each species has unique habitat preferences and phenology [15]. Most canine tick infestations are acquired from natural outdoor environments with the notable exception of *R. sanguineus*, an endophilic tick inhabiting homes and kennels [15]. Although published records are incomplete, the geographical distribution of *Ixodes* spp. in North America and Europe and of *A. americanum* in North America has dramatically expanded in recent decades [16–19], leading to an increased need for straightforward, robust tick control strategies for dogs in many different regions of the globe. The tick studies presented in this special issue demonstrate that lotilaner treatment readily eliminates infestation with three key European species (*I. ricinus*, *D. reticulatus* and *R. sanguineus*) and the four major North American species (*A. americanum*, *D. variabilis*, *I. scapularis* and *R. sanguineus*), and that all of the different tick species continued to be killed at a very high efficacy throughout the month following treatment [20, 21]. Detailed studies with *I. ricinus* revealed the ticks present at the time of treatment were killed within 4–8 h of initial administration, and newly acquired *I. ricinus* ticks were killed within 12 h of infestation throughout the 35-day study [22]. Both the rapid onset of action and sustained speed of kill are important; systemic acaricides have been associated with decrease in or complete prevention of transmission of tick-borne disease agents in several studies, including those evaluating the ability of acaricides to prevent tick-borne infection with *Borrelia burgdorferi*, *Anaplasma phagocytophilum*, *Ehrlichia canis* and *Babesia canis* [23–25].

Dog ownership provides many benefits to human health, including encouraging opportunities for exercise, reducing the impact of stressful life events, and developing capacity for empathy, especially in children [1]. However, tick and flea infestations directly threaten both human and canine health. Infestations are at best a nuisance to the owner, dog, and veterinarian alike that often discourages owners from keeping pets indoors or even keeping a pet at all. At worst, these arthropods and the infections they transmit can severely compromise the health of dogs and people. As we forge ever closer bonds with companion animals, insuring dogs and the homes we share with them remain free of ticks and fleas becomes increasingly important. By providing safe, fast, and effective flea control and broad-spectrum tick efficacy for up to 35 days after administration, lotilaner, a novel insecticidal and acaricidal compound, allows us to achieve this goal of protecting dogs from ticks and fleas and protecting the special relationship between dogs and their owners. French translation of the article is available in Additional file 1.

## Additional file

Additional file 1: French translation of the article. (PDF 33 kb)
