# Supplementary material for: Lotilaner - a novel systemic tick and flea control product for dogs
Source: Parasit Vectors. 2017 Nov 1;10:539. doi: 10.1186/s13071-017-2471-3 (PMC5664799; doi:10.1186/s13071-017-2471-3)
Supplement: Additional file 1: — French translation of the article. (PDF 33 kb) [file 13071_2017_2471_MOESM1_ESM.pdf]

Le lotilaner, un nouvel antiparasitaire systémique actif contre les puces et les tiques chez le chien

Susan E. Little[1]

[1]Center for Veterinary Health Sciences, Oklahoma State University, Stillwater, Oklahoma, 74074 États-Unis

Correspondance : susan.little@okstate.edu

Il est essentiel d'exercer un contrôle efficace et sûr des tiques et puces pour préserver la santé et le bien-être des animaux de compagnie et des personnes qui partagent leur quotidien. Réduire le risque inhérent aux infestations par ces arthropodes protège le chien et l'homme, non seulement contre les lésions qu'ils provoquent, mais aussi, très souvent, contre les infections graves qu'ils transmettent. Un autre bénéfice, peut-être aussi important, du contrôle efficace des tiques et puces est la préservation du lien qui unit l'homme à l'animal. Les antiparasitaires les plus récents, tels que le lotilaner, la nouvelle isoxazoline décrite dans cette publication, permettent aux vétérinaires et aux propriétaires de lutter, de façon simple et efficace, contre ce fléau pour que tout le stress lié aux infestations du foyer ne soit plus qu'un mauvais souvenir. Supprimer les tiques et puces de la relation homme-animal renforce ce lien même qui les unit, avec à terme un réel bénéfice sur de nombreux aspects de la santé publique, physique comme mentale [1]. Développer le lien entre l'homme et l'animal et protéger la santé humaine et canine font partie des principales raisons pour lesquelles le Companion Animal Parasite Council (capcvet.org) et l'European Scientific Counsel on Companion Animal Parasites (esccap.org) recommandent le contrôle systématique des infestations par les tiques et les puces chez le chien.

Cette publication spéciale présente un ensemble d'études approfondies concernant l’innocuité, l'efficacité et les performances du lotilaner, un nouvel insecticide et acaracide systémique de la classe des isoxazolines. Le lotilaner a été spécifiquement développé par Elanco pour l'animal de compagnie afin d'éliminer rapidement les infestations par les tiques et les puces chez le chien. Les rapports des études sur la pharmacocinétique et l'innocuité du lotilaner montrent son absorption rapide et l'absence de tout effet pathologique ou lié au traitement, même en cas d'administration de doses élevées pendant plusieurs mois [2, 3]. L'excellent profil de sécurité du

lotilaner et d'autres isoxazolines est un autre avantage de l'utilisation des insecticides et acaricides récemment développés pour les animaux de compagnie. Ainsi, des produits comme le lotilaner ont largement supplanté d'autres composés historiques, plus toxiques, qui ont souvent été interdits tels que les organochlorés, les organophosphorés et les carbamates [4, 5].

Ctenocephalides felis reste l'espèce de puce prépondérante chez les animaux de compagnie, et ce dans le monde entier. Malgré le développement depuis plusieurs décennies d'un large éventail de produits permettant un contrôle efficace des puces, les infestations restent un problème majeur en santé canine, aggravé par la présence de populations de *C. felis* résistantes aux insecticides [6]. Les études expérimentales d'infestations par des puces décrites dans cette publication spéciale montrent toute l'efficacité et la rapidité d'action du lotilaner, un nouvel insecticide systémique, qui commence à tuer les puces dans les 2 heures suivant son administration et reste actif (dans les 4 heures) contre de nouvelles infestations pendant au moins 35 jours après l'administration initiale [7, 8]. Des études de terrain ont confirmé que les propriétaires peuvent s'attendre, en utilisant le lotilaner, à la disparition des puces et à une diminution significative des signes de dermatite par allergie aux piqûres de puces (DAPP) [9, 10]. L'effet létal rapide, l'élimination totale des infestations et la prévention de nouvelles infestations sont essentiels pour minimiser les manifestations de DAPP car, même un tout petit nombre de piqûres peut entraîner une recrudescence des signes cliniques [11]. De plus, les comprimés de lotilaner ont été facilement acceptés par les chiens. Lors d'une étude comparative menée en conditions réelles d'infestation, ils se sont aussi avérés plus efficaces contre les puces que le fipronil [10]. Cette grande acceptabilité est importante : malgré les nombreuses avancées en termes d'innocuité et d'efficacité des antiparasitaires, le défaut d'observance reste un obstacle important au contrôle des puces chez le chien [12-14].

Plusieurs espèces de tiques sont susceptibles d'infester le chien, notamment *Rhipicephalus sanguineus* (*sensu lato*) partout dans le monde, *Ixodes ricinus* et *Dermacentor reticulatus* en Europe et *Amblyomma americanum, Dermacentor variabilis* et *Ixodes scapularis* en Amérique du Nord. Les préférences en termes d'habitat et la phénologie sont propres à chaque espèce [15]. Les tiques infestent le plus souvent le chien lorsqu'il se trouve à l'extérieur, dans un environnement naturel. *R. sanguineus* constitue néanmoins une exception notable : cette espèce endophile est présente dans les foyers et les chenils [15]. Bien que les données publiées soient incomplètes, la répartition géographique d'*Ixodes* spp. en Amérique du Nord et en Europe et d'*A. americanum* en Amérique du Nord s'est considérablement étendue ces dernière décennies [16-19]. Ainsi, le besoin de méthodesde contrôle simples et efficaces n'a jamais été aussi important pour le chien dans de nombreuses régions du monde. Les études menées sur les tiques présentées dans cette publication spéciale montrent que

le traitement par le lotilaner élimine facilement les infestations par trois espèces européennes majeures (*I. ricinus*, *D. reticulatus* et *R. sanguineus*) et par les quatre espèces les plus répandues en Amérique du Nord (*A. americanum*, *D. variabilis*, *I. scapularis* et *R. sanguineus*). Ces études ont également établi la persistance d'un effet acaricide particulièrement efficace sur chacune de ces espèces durant tout le mois suivant le traitement [20, 21]. Les études approfondies concernant *I. ricinus* ont révélé que les tiques présentes au moment du traitement ont été tuées dans les 4 à 8 heures suivant l'administration initiale et que les tiques *I. ricinus* nouvellement acquises étaient tuées dans les 12 heures suivant l'infestation tout au long des 35 jours de l'étude [22]. La rapidité d'action et la rémanence de l'effet acaricide sont toutes deux importantes. Plusieurs études ont associé le traitement par des acaricides systémiques à une diminution ou à un blocage total des maladies vectorielles, y compris celles évaluant la capacité des acaricides à prévenir la transmission des infections par *Borrelia burgdorferi*, *Anaplasma phagocytophilum*, *Ehrlichia canis* et *Babesia canis* [23-25].

La présence d'un chien au foyer est bénéfique à plus d'un titre : encouragement à faire plus d'exercice, réduction de l'impact des événements stressants, développement de l'empathie, en particulier chez l'enfant [1]. Pourtant, les infestations par les puces et les tiques menacent directement la santé de l'homme et du chien. Les infestations sont, au mieux, une simple nuisance pour le propriétaire, le chien et leur vétérinaire qui incite souvent les maîtres à ne pas garder leur animal à l'intérieur, voire dans certains cas à ne pas prendre d'animaux du toutr. Dans le pire des cas, ces arthropodes et les infections qu'ils transmettent peuvent gravement compromettre la santé de l'homme et du chien. Il devient d'autant plus important de protéger les chiens et leurs foyers des tiques et des puces que le lien qui unit l'homme à l'animal est de plus en plus fort. En conférant un contrôle sûr, rapide et efficace des puces et un large spectre d'activité contre les tiques jusqu'à 35 jours après son administration, le lotilaner, un nouvel insecticide et acaricide, permet d'atteindre cet objectif de protection contre les tiques et les puces et de préserver ainsi le lien, si précieux, qui unit un chien à son propriétaire.

Déclaration d'éthique et autorisation de participation

Sans objet.

Autorisation de publication

Sans objet.

Disponibilité des données et supports

Sans objet.

Conflit d'intérêts

Elanco a ponctuellement versé des honoraires et remboursé des frais de déplacement à l'auteur au cours des 5 dernières années.

Financement

Sans objet.

Affiliation de l'auteur

[1]Center for Veterinary Health Sciences, Oklahoma State University, Stillwater, Oklahoma, 74074 États-Unis.

Références bibliographiques

1. Barker SB, Wolen AR. The benefits of human-companion animal interaction: a review. J Vet Med Educ. 2008;35:487–495.
2. Kuntz EA, Kammanadiminti S. Safety evaluation of lotilaner in dogs after oral administration as flavoured chewable tablets (Credelio™). Parasit Vectors. 2017; présente publication.
3. Toutain CE, Seewald W, Jung M. The intravenous and oral pharmacokinetics of lotilaner in dogs. Parasit Vectors. 2017; présente publication.
4. Wismer T1, Means C. Toxicology of newer insecticides in small animals. Vet Clin North Am Small Anim Pract. 2012;42:335-47.
5. Beugnet F, Franc M. Insecticide and acaricide molecules and/or combinations to prevent pet infestation by ectoparasites. Trends Parasitol. 2012;28:267-79.
6. Rust MK. Insecticide resistance in fleas. Insects. 2016;7:10.
7. Cavalleri D, Murphy M, Seewald W, Drake J, Nanchen S. Assessment of the speed of flea kill of lotilaner (Credelio™) throughout the month following oral administration to dogs. Parasit Vectors. 2017; présente publication.
8. Cavalleri D, Murphy M, Seewald W, Drake J, Nanchen S. Assessment of the onset of lotilaner (Credelio™) speed of kill of fleas on dogs. Parasit Vectors. 2017; présente publication.

9. Karadzovska D, Chappell K, Coble S, Murphy M, Cavalleri D, Wiseman S, et al. A randomized, controlled field study to assess the efficacy and safety of lotilaner flavored chewable tablets (Credelio™) in eliminating fleas in client-owned dogs in the USA. Parasit Vectors. 2017; présente publication.
10. Cavalleri D, Murphy M, Seewald W, Drake J. A randomized, blinded, controlled field study to assess the efficacy and safety of lotilaner tablets (Credelio™) in controlling fleas in client-owned dogs in Europe. Parasit Vectors. 2017; présente publication.
11. Carlotti DN, Jacobs DE. Therapy, control and prevention of flea allergy dermatitis in dogs and cats. Vet Dermatol. 2000;11:83–98.
12. Dryden MW: Flea and tick control in the 21st century: challenges and opportunities. Vet Dermatol. 2009;20:435–440.
13. Coles TB, Dryden MW. Insecticide/acaricide resistance in fleas and ticks infesting dogs and cats. Parasit Vectors. 2014;7:8.
14. Halos L, Beugnet F, Cardoso L, Farkas R, Franc M, Guillot J, et al. Flea control failure? Myths and realities. Trends Parasitol. 2014;30:228-33.
15. Dryden MW, Payne PA. Biology and control of ticks infesting dogs and cats in North America. Vet Ther. 2004;5:139-54.
16. Medlock JM, Hansford KM, Bormane A, Derdakova M, Estrada-Peña A, George JC, et al. Driving forces for changes in geographical distribution of *Ixodes ricinus* ticks in Europe. Parasit Vectors. 2013;6:1.
17. Barrett AW, Noden BH, Gruntmeir JM, Holland T, Mitcham JR, Martin JE, et al. County scale distribution of *Amblyomma americanum* (Ixodida: Ixodidae) in Oklahoma: Addressing local deficits in tick maps based on passive reporting. J Med Entomol. 2015;52:269-73.
18. Khatchikian CE, Prusinski MA, Stone M, Backenson PB, Wang IN, Foley E, et al. Recent and rapid population growth and range expansion of the Lyme disease tick vector, *Ixodes scapularis*, in North America. Evolution. 2015;69:1678-89.
19. Christenson M, Lee X, Larson S, Johnson DH, Jensen J, Meller M, Paskewitz S. Occurrence of *Amblyomma americanum* (Acari: Ixodidae) and human infection with *Ehrlichia chaffeensis* in Wisconsin, 2008–2015. J Med Entomol. 2016; doi: 10.1093/jme/tjw218
20. Cavalleri D, Murphy M, Gorbea RL, Seewald W, Drake J, Nanchen S. Laboratory evaluations of the immediate and sustained effectiveness of lotilaner (Credelio™) against three common species of ticks affecting dogs in Europe. Parasit Vectors. 2017; présente publication.

21. Murphy M, Garcia R, Karadzovska D, Cavalleri D, Snyder D, Seewald W, et al. Laboratory evaluations of the immediate and sustained efficacy of lotilaner (Credelio™) against four common species of ticks affecting dogs in North America. Parasit Vectors. 2017; présente publication.
22. Murphy M, Cavalleri D, Seewald W, Drake J, Nanchen S. Laboratory evaluation of the speed of kill of lotilaner (Credelio™) against *Ixodes ricinus* ticks on dogs. Parasit Vectors. 2017; présente publication.
23. Taenzler J, Liebenberg J, Roepke RK, Heckeroth AR. Prevention of transmission of *Babesia canis* by *Dermacentor reticulatus* ticks to dogs treated orally with fluralaner chewable tablets (Bravecto™). Parasit Vectors. 2015;8:305.
24. Honsberger NA, Six RH, Heinz TJ, Weber A, Mahabir SP, Berg TC. Efficacy of sarolaner in the prevention of *Borrelia burgdorferi* and *Anaplasma phagocytophilum* transmission from infected *Ixodes scapularis* to dogs. Vet Parasitol. 2016;222:67-72.
25. Jongejan F, Crafford D, Erasmus H, Fourie JJ, Schunack B. Comparative efficacy of oral administrated afoxolaner (NexGard™) and fluralaner (Bravecto™) with topically applied permethrin/imidacloprid (Advantix(®)) against transmission of *Ehrlichia canis* by infected *Rhipicephalus sanguineus* ticks to dogs. Parasit Vectors. 2016;9:348.
